# QSAR-Assisted Virtual Screening of Lead-Like Molecules from Marine and Microbial Natural Sources for Antitumor and Antibiotic Drug Discovery

**DOI:** 10.3390/molecules20034848

**Published:** 2015-03-17

**Authors:** Florbela Pereira, Diogo A. R. S. Latino, Susana P. Gaudêncio

**Affiliations:** 1 Centro de Química Fina e Biotecnologia (CQFB)/LAQV-REQUIMTE, Departamento de Química, Faculdade de Ciências e Tecnologia, Universidade Nova de Lisboa Campus Caparica, Caparica 2829-516, Portugal; E-Mails: dalatino@fc.ul.pt (D.A.R.S.L.); s.gaudencio@fct.unl.pt (S.P.G.); 2 Centro de Ciências Moleculares e Materiais (CCMM), Departamento de Química e Bioquímica, Faculdade de Ciências, Universida Lisboa, Campo Grande, Lisboa 1749-016, Portugal

**Keywords:** quantitative structure-activity relationships (QSAR), semi-empirical quantum-chemical descriptors, marine natural products, microbial natural products, antibiotic, antitumor, drug discovery

## Abstract

A Quantitative Structure-Activity Relationship (QSAR) approach for classification was used for the prediction of compounds as active/inactive relatively to overall biological activity, antitumor and antibiotic activities using a data set of 1746 compounds from PubChem with empirical CDK descriptors and semi-empirical quantum-chemical descriptors. A data set of 183 active pharmaceutical ingredients was additionally used for the external validation of the best models. The best classification models for antibiotic and antitumor activities were used to screen a data set of marine and microbial natural products from the AntiMarin database—25 and four lead compounds for antibiotic and antitumor drug design were proposed, respectively. The present work enables the presentation of a new set of possible lead like bioactive compounds and corroborates the results of our previous investigations. By other side it is shown the usefulness of quantum-chemical descriptors in the discrimination of biologically active and inactive compounds. None of the compounds suggested by our approach have assigned non-antibiotic and non-antitumor activities in the AntiMarin database and almost all were lately reported as being active in the literature.

## 1. Introduction

Natural products (NPs), or synthetic products inspired by NPs, have been the single most productive source leads for the development of drugs. In fact, more than half of the approved drugs from 1981 to 2010 were based on NPs [1]. From the detection of the antibiotics properties of penicillin by Fleming in 1929, through “the Golden Age of Antibiotics” when almost all groups of important antibacterial antibiotics (e.g., tetracyclines cephalosporins, aminoglycosides, and macrolides) were discovered and into the late 20th century, the promise of unprecedented structural diversity and potent biological activity from microbial secondary metabolites remained a powerful force driving to pharmaceutical discovery. Microbes are the most prolific source of bioactive metabolites with a rate of 44%–46% comparing with the overall rate of 20%–25% for all NPs sources [2]. Furthermore bacteria are an exceptional source of small molecule chemical diversity. Although the terrestrial bacteria had been more studied in the past, a growing interest in its distribution and ecological role in the marine environment has been observed. The oceans are a highly complex microbiological environment with typical microbial abundances of 10^6^ and 10^9^ per ml in seawater and ocean-bottom sediments, respectively [3]. For instances, the rare and complex densely functionalized γ-lactam-β-lactone pharmacophore, salinosporamide A (trade name Marizomib, NPI-0052), from the seawater-obligate marine actinomycete bacteria, *Salinispora tropica*, is heading for phase II clinical trials against cancer, but may not be a clinical candidate due to the demise of Nereus Pharmaceuticals in late 2013 [4]. As marine organisms are an important source of structurally diverse and biologically active secondary metabolites, a new branch of NPs chemistry had been fully established—Marine Natural Products (MNPs). The current success rate of discovery from the marine world: seven clinically approved drugs [5], (four anticancer, one antiviral, one pain control, and one hypertriglyceridemia), from 22,000 discovered molecular entities [6] (e.g., one drug per 3140 NPs described) approximately 1.7- to 3.3-fold better than the industry average (one in 5000–10,000 tested compounds) [7].

Currently, there are facilities for high-throughput screening available both in academic labs as well as in drug pharmaceutical companies, but the cost of random screening for very large collections of compounds can nevertheless be prohibitive. Thus it makes sense to use chemoinformatics approaches for the virtual screening of the most probable active compounds. Although, in the last years few computational approaches have been applied for *in silico* screening of NPs [8,9,10,11,12,13,14,15,16]. This is a field that can be significantly improved by the modeling of data from large databases containing information relatively to biological activities, which are becoming available to the scientific community. AntiMarin [17] is a good example of a powerful database, which contains approximately 50,000 compounds from marine macroorganisms and both marine and terrestrial microorganisms. This database is, in fact, the result of a fusion between AntiBase [18] (a database of all terrestrial and marine microbial NPs) and MarinLit [6] (a database of MNPs literature). Therefore, besides MNPs we will refer the NP derived from microbial source as microbial natural products M_b_NPs, accounting for those of terrestrial origin.

In the last years quantum-chemical descriptors have been used with success in a great variety of SPR/QSPR and SAR/QSAR applications from prediction of chemical reactivities, physicochemical properties, partition coefficients, chromatographic retention indexes, and biological activities [19]. In the context of QSAR studies quantum-chemical descriptors, e.g., net atomic charges, HOMO and LUMO energies, hardness, chemical potential, electrophilicity index, have been shown to be useful in the estimation of various biological activities, for example in studies related with estimation of toxicity, mutagenicity and carcinogenicity as well as in studies of their mechanisms of action [20,21,22]. The usefulness of these descriptors was also been demonstrated by the estimation and study of antioxidant, antitumor or antibacterial activities. Theoretical studies of the electronic properties and chemical reactivity of quercetin [23], catechin and epicatechin [24] were as well reported. In other studies, the electrophilicity index was related to the ability of the NPs isoprekinamycin, kinamycins and lomaiviticin A acting as antitumor and antibacterial agents [25], to study the antileukaemic activity of phenol [26], and in suppression of breast cancer by chemical modulation of vulnerable zinc fingers in estrogen receptors [27]. Quantum-chemical descriptors have also been used in the analysis of the relations between the structural properties and the antitumoral activity of synthetic chalcones [28]. The antimicrobial activity of *N*-phenylbenzamide derivatives [29] and the antiparasitic activity of the nifurtimox analogues [30] are also correlated with electrophilicity index. In our group the combination of quantum chemical and empirical descriptors were used in the past to establish QSRR models for the estimation of Mayr electrophilicity with great success [31].

The present study focuses on the application of machine learning (ML) techniques to exploit lead-like molecules *en route* to antitumor and antibiotic drugs from 418 MNPs and M_b_NPs (extracted from the AntiMarin database, AntiMarin set). The models were developed using 1746 active and non-active compounds from the PubChem database. State-of-the-art ML algorithms, such as Support Vector Machines (SVMs), Random Forests (Rfs) and Classification Tree (CTs), were compared to predict the two classes (*i.e.*, active and non-active compounds) in the following classification tasks: (1) the overall biological activity; (2) the antitumor activity; and (3) the antibiotic activities. For each task three models were built using: 232 CDK descriptors, eight semi-empirical quantum-chemical descriptors calculated by the PM6 method (PM6 descriptors) and finally using simultaneously CDK descriptors and PM6 descriptors. Using internal (cross-validation and out-of-bag estimation on the training set) and external validation (on two external data sets from PubChem, test set I comprises of 863 compounds and test set II comprises of 183 active pharmaceutical ingredients) tests all of these QSAR models have been validated. In addition, a further test set (AntiMarin set) extracted from the AntiMarin database was used but not with the main purpose of external model validation. The results obtained with these three approaches were compared with our recently published work using only CDK descriptors [15].

## 2. Results and Discussion

### 2.1. Establishment of QSAR Classification Models

The results for internal validation (ten-fold cross-validation with SVM and out-of-bag estimation with Rf on the training set) and external validation (on test set I) for each task (*i.e.*, overall biological activity, antitumor and antibiotic activities) are presented in Table 1, Table 2 and Table 3.

**Table 1:** Comparison of different ML techniques and descriptors for building overall biological activity classification models.

		SVM ^a^	CT	Rf ^b^
Training set/Test Set I/AntiMarin Set				
Descriptors	Class Size	Correct Predictions		
		Sensitivity ^c^		
		Specificity ^d^		
		G-mean ^e^		
**CDK ^f^**	Active 1612/798/315 Non-active 134/65/103	Active	Active	Active
		1199/646/242	1599/782/312	1114/566/251
		Non-active	Non-active	Non-active
		79/34/30	32/5/1	93/53/33
		0.74/0.81/0.77	0.99/0.98/0.99	0.69/0.71/0.80
		0.59/0.52/0.29	0.24/0.08/0.01	0.69/0.82/0.32
		0.66/0.65/0.47	0.49/0.27/0.10	0.69/0.76/0.51
**PM6 ^g^**		Active	Active	Active
		1043/535/252	1603/795/311	1061/532/248
		Non-active	Non-active	Non-active
		60/40/23	14/3/1	63/38/23
		0.65/0.67/0.80	0.99/1.00/0.99	0.66/0.67/0.79
		0.45/0.62/0.22	0.10/0.05/0.01	0.47/0.58/0.22
		0.54/0.64/0.42	0.32/0.21/0.10	0.56/0.62/0.42
**CDK+PM6**		Active	Active	Active
		1244/649/236	1598/764/305	1124/577/256
		Non-active	Non-active	Non-active
		75/37/29	49/13/3	89/50/27
		0.77/0.81/0.75	0.99/0.96/0.97	0.70/0.72/0.81
		0.56/0.57/0.28	0.37/0.20/003	0.66/0.77/0.26
		0.66/0.68/0.46	0.60/0.44//0.17	0.68/0.75/0.46

Notes: ^a^ Ten-fold cross-validation; ^b^ Out-of-bag; ^c^ The ratio of true positives to the sum of true positives and false negatives; ^d^ The ratio of true negatives to the sum of true negatives and false positives; ^e^ The square root of the product of sensitivity and specificity; ^f^ QSAR model built using 232 CDK descriptors; ^g^ QSAR model built using 8 quantum-mechanical descriptors calculated by the semi-empirical method PM6.

**Table 2:** Comparison of different ML techniques and descriptors for building antitumor activity classification models.

		SVM ^a^	CT	Rf ^b^
Training set/Test Set I/AntiMarin Set				
Descriptors	Class Size	Correct Predictions		
		Sensitivity ^c^		
		Specificity ^d^		
		G-mean ^e^		
**CDK ^f^**	Active 880/438/58 Non-active 866/425/360	Active	Active	Active
		707/352/15	735/354/12	763/366/22
		Non-active	Non-active	Non-active
		556/291/220	631/296/245	675/348/247
		0.80/0.80/0.26	0.84/0.81/0.17	0.87/0.84/0.38
		0.64/0.68/0.61	0.73/0.70/0.68	0.78/0.82/0.69
		0.72/0.74/0.40	0.78/0.75//0.34	0.82/0.83/0.51
**PM6 ^g^**		Active	Active	Active
		533/256/23	663/306/30	543/273/22
		Non-active	Non-active	Non-active
		511/255/209	475/204/157	507/247/188
		0.61/0.58/0.40	0.75/0.70/0.52	0.62/0.62/0.38
		0.59/0.60/0.58	0.55/0.48/0.44	0.59/0.58/0.52
		0.60/0.59/0.48	0.64/0.58/0.48	0.60/0.60/0.44
**CDK+PM6**		Active	Active	Active
		689/344/12	735/354/12	763/374/18
		Non-active	Non-active	Non-active
		620/313/244	631/296/245	679/344/256
		0.78/0.79/0.21	0.84/0.81/0.17	0.87/0.85/0.31
		0.72/0.74/0.68	0.73/0.70/0.68	0.78/0.81/0.71
		0.75/0.76/0.37	0.78/0.75//0.34	0.82/0.83/0.47

Notes: ^a^ Ten-fold cross-validation; ^b^ Out-of-bag; ^c^ The ratio of true positives to the sum of true positives and false negatives; ^d^ The ratio of true negatives to the sum of true negatives and false positives; ^e^ The square root of the product of sensitivity and specificity; ^f^ QSAR model built using 232 CDK descriptors; ^g^ QSAR model built using 8 quantum-mechanical descriptors calculated by the semi-empirical method PM6.

An additional test set (AntiMarin set) extracted from the AntiMarin database was also used but not with the main purpose of external model validation. The AntiMarin data set was screened by the developed models to find lead-like molecules *en route* to antitumor and antibiotic drugs, which, indeed, is the core of the presented. Moreover, the inclusion of quantum-chemical descriptors, which have been long used in QSAR studies in biochemistry, allows the opportunity of evaluating their importance in the modeling of overall biological, antitumor, and antibiotic activities.

The best model was accomplished for each task with Rfs that showed a better performance when compared to a single CT and SVMs in the prediction of the overall biological, antitumor, and antibiotic activities for test set I using both the 232 CDK descriptors and the 8 PM6 descriptors, taking into account the value of the G-mean (Table 1, Table 2 and Table 3). The predictions obtained from these models using simultaneously the CDK and PM6 descriptors are slightly better than those using only the CDK descriptors, Table 1, Table 2 and Table 3. However, for all tasks, the performance of the models built only with PM6 descriptors is worse than that obtained using only the CDK descriptors. As expected the predictive power (taking into account the value of the G-mean for test set I) of CT is lower than that obtained for the other two ML techniques for almost all the activity models in the different approaches (*i.e.*, CDK, PM6, and CDK+PM6 models). However a CT has the advantage of establish a few simple rules that can provide insights into the properties drug profile with a given biological activity.

**Table 3:** Comparison of different ML techniques and descriptors for building antibiotic activity classification models.

		SVM ^a^	CT	Rf ^b^
Training set/Test Set I/AntiMarin Set				
Descriptors	Class Size	Correct Predictions		
		Sensitivity ^c^		
		Specificity ^d^		
		G-mean ^e^		
**CDK ^f^**	Active 642/326/228 Non-active 1104/537/190	Active	Active	Active
		483/236/159	514/246/150	535/268/163
		Non-active	Non-active	Non-active
		997/485/126	1049/484/123	1045/502/118
		0.75/0.72/0.70	0.80/0.76/0.66	0.83/0.82/0.71
		0.90/0.90/0.66	0.95/0.90/0.65	0.95/0.93/0.62
		0.82/0.81/0.68	0.87/0.83//0.65	0.89/0.88/0.67
**PM6 ^g^**		Active	Active	Active
		440/227/144	182/83/48	432/229/136
		Non-active	Non-active	Non-active
		642/302/64	995/484/153	668/307/66
		0.69/0.70/0.63	0.28/0.26/0.21	0.67/0.70/0.60
		0.58/0.56/0.34	0.90/0.90/0.81	0.60/0.57/0.35
		0.63/0.63/0.46	0.50/0.48/0.41	0.64/0.63/0.46
**CDK+PM6**		Active	Active	Active
		485/236/161	514/246/150	536/266/161
		Non-active	Non-active	Non-active
		994/485/124	1049/484/123	1047/504/120
		0.76/0.72/0.71	0.80/0.76/0.66	0.83/0.82/0.71
		0.90/0.90/0.65	0.95/0.90/0.65	0.95/0.94/0.63
		0.82/0.81/0.68	0.87/0.83//0.65	0.89/0.88/0.67

Notes: ^a^ Ten-fold cross-validation; ^b^ Out-of-bag; ^c^ The ratio of true positives to the sum of true positives and false negatives; ^d^ The ratio of true negatives to the sum of true negatives and false positives; ^e^ The square root of the product of sensitivity and specificity; ^f^ QSAR model built using 232 CDK descriptors; ^g^ QSAR model built using 8 quantum-mechanical descriptors calculated by the semi-empirical method PM6.

Moreover, the best Rfs models were further validated using a test set II comprising 183 active pharmaceutical ingredients (APIs) also extracted from PubChem. The results are shown in Table 4. Although, the predictions obtained for the test set II using the CDK and PM6 descriptors are, in general, better than those obtained using only the CDK or PM6 descriptors, it appears that the importance of the PM6 descriptors is greater, for this data set, than that for the test set I. These results will be discussed in leading topics presented in the following sections. 

**Table 4:** Rfs predictions of the overall biological activity, antitumor and antibiotic activities for the test set II.

		CDK ^a^	PM6 ^b^	CDK+PM6
Model	Class Size	Correct Predictions		
		Sensitivity ^c^		
		Specificity ^d^		
		G-mean ^e^		
Overall	Active 183 Non-active 0	Active	Active	Active
		114	148	121
		Non-active	Non-active	Non-active
		69	35	62
		0.62	0.81	0.66
		na ^f^	na	na
		0.79	0.90	0.81
Antitumor	Active 68 Non-active 115	Active	Active	Active
		55	41	56
		Non-active	Non-active	Non-active
		26	41	26
		0.81	0.60	0.82
		0.23	0.36	0.23
		0.43	0.46	0.43
Antibiotic	Active 29 Non-active 154	Active	Active	Active
		17	27	18
		Non-active	Non-active	Non-active
		150	68	149
		0.59	0.93	0.62
		0.97	0.44	0.97
		0.76	0.64	0.77

Notes: ^a^ QSAR model built using 232 CDK descriptors; ^b^ QSAR model built using 8 quantum-mechanical descriptors calculated by the semi-empirical method PM6; ^c^ The ratio of true positives to the sum of true positives and false negatives; ^d^ The ratio of true negatives to the sum of true negatives and false positives; ^e^ The square root of the product of sensitivity and specificity; ^f^ Not applicable.

**Figure 1:**
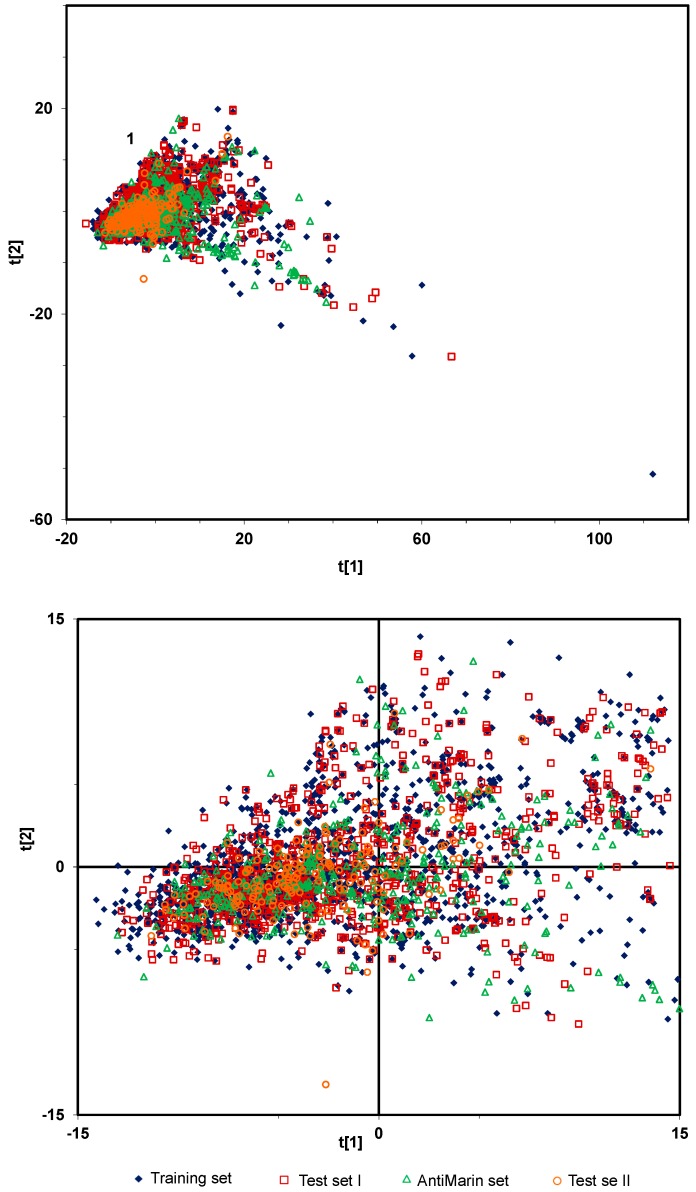
PCA score plot derived from CDK and PM6 descriptors, PC1 (t[1]) *versus* PC2 (t[2]). The bottom plot is the amplification of the cluster highlighted as 1.

One of the most-widely used multivariate exploratory techniques is the Principal Component Analysis (PCA) [32,33]. It is able to detect similarities among data sets of compounds providing a statistically reliable criterion to classify the compounds upon their different physicochemical property pattern against different biological activities. To obtain a general impression of all data set that were used for modeling (*i.e.*, training, test set I, test set II from PubChem and AntiMarin set), a PCA model was derived from the CDK and PM6 descriptors, which describe a range of electronic, steric, geometrical, and quantum properties of the compounds. The first two components (respectively accounting for 37% and 8% of the variance in the descriptor matrix) are shown in Figure 1. Results obtained from the PCA score plots in Figure 1 shows that no clearly defined separation exists between the four data sets based upon the major source of variation within their electronic, steric, geometrical, and quantum property profiles.

Moreover, approximately 93% of all the compounds in the four data sets are well clustered together in the cluster highlighted as 1 (Figure 1). Therefore, the predictions for all the test sets may be considered reliable since their compounds fall within a chemical-space domain defined by the training set compounds used to build the QSAR models.

#### 2.1.1. Overall Biological Activity Model

For the overall biological activity models the data are imbalanced in what concerns the active and non-active classes, and hence cause a problem for the CT method, which is not able to balance the two classes, as has been done with the other two methods (*i.e.*, by adjusting the weight of each class). As it was expected the overall CT biological models using the CDK, PM6 and CDK+PM6 approaches show poor predictions accuracy—a G-mean of 0.27, 0.21 and 0.44 for test set I, respectively, Table 1. However, it appears to be an improve in the prediction power of the CT model with inclusion of PM6 descriptors since the CT overall biological model using only the CDK descriptors shows a lower prediction accuracy (a G-mean of 0.27 for test set I).

With the overall biological activity models, we intended to improve the precision of lead-like profiling by compensating for the lack of bioactivity records in the 418 compounds extracted from the AntiMarin database as we had done in our previous work and as well as compare the selected lead bioactive compounds proposed by the two approaches. In the AniMarin database, as almost all databases, each molecule has been tested for only a few activities chosen by the researchers or grant programs, so there are almost certainly many bioactive molecules that lack an activity record. There are probably compounds that were classified as non-active against one given biological activity only because they have never been tested against that biological assay. Therefore, we would expect that the difficulty with this model was actually the false positives (FPs) for the AntiMarin set. In fact, we obtained a large number of FPs for the AntiMarin set, as shown by the low specificity value of 0.26 obtained for the best model (Rf using CDK+PM6) compared with the high specificity value of 0.77 obtained for the test set I, Table 1. The analysis of the results obtained from the best Rf model has shown that there are 76 and 15 FPs for the AntiMarin set and test set I, respectively. For these FPs, it was obtained an average probability of being active (Avg.Prob_active_) in the best Rf model of 0.66 (30 FPs have a Avg.Prob_active_ ≥ 0.7), and 0.61 (2 FPs have a Avg.Prob_active_ ≥ 0.7) for the AntiMarin set and test set I, respectively.

Many authors have tried to rationalize the drug-like and lead-like nature of compounds, Waldmann *et al.* [10] suggested through a statistical analysis of the structural classification of NPs that the lead-like molecules must have a scaffold with two, three, or four rings, and their van der Waals volumes must match the lower end of the majority of the protein cavities (*i.e.*, van der Waals volume between 300 and 800 Å^3^). Indeed, as recently reported [15], the analysis of the active and non-active profiles of the van der Waals volume of the training set and test sets from PubChem and AntiMarin set from AntiMarin revealed there is a correlation between active compounds and 3-, 4-ringed compounds with a van der Waals volume between 300 and 800 Å^3^. In the AntiMarin set, there are 87 compounds with these specifications. From those only 68 compounds (*i.e.*, approximately 78%) are active as compared with approximately 96% of active compounds from the test set I compounds (PubChem) with the same specifications. Moreover, from those with the same specifications 59 and 14 compounds were predicted as true positives (TPs) and FPs with an Avg.Prob_active_ of 0.73 and 0.70, respectively, using the best Rf model for the AntiMarin set. In the test set II, approximately 75% of the APIs with these specifications were predicted to be active by the best Rf model. The energy of the highest occupied molecular orbital (ε_HOMO_) is the first and second descriptor selected to build the overall biological activity classification tree models using PM6 and CDK+PM6 descriptors, respectively, see Table 5.

**Table 5:** Comparison of descriptors selected with descriptor importance using to build QSAR models.

Models			CDK Descriptors	PM6 Descriptors	CDK+PM6 Descriptors
**Overall biological activity**	SVM ^a^		**20D**: ALogp2; BCUTc-1l; BCUTp-1h; PPSA-2; DPSA-3; FPSA-3; TPSA; Wlambda2.unity; Weta1.unity; ATSc3; SCH-5; SP-6; VP-7; khs.ssCH2; khs.dsCH; khs.sssCH; khs.aaaC; khs.sNH2; MDEC-33; TopoPSA	**8D**: ε_HOMO_; ε_LUMO_; Mulliken electronegativity (χ); Parr & Pople absolute hardness; Schuurmann MO shift alpha; hardness (η); chemical potential (μ); electrophilicity index (ω)	**21D**: ALogp2; BCUTc-1l; BCUTp-1h; PPSA-2; DPSA-3; FPSA-3; TPSA; Wlambda2.unity; Weta1.unity; ATSc3; SCH-5; SP-6; VP-7; khs.ssCH2; khs.dsCH; khs.sssCH; khs.aaaC; khs.sNH2; MDEC-33; TopoPSA; ε_HOMO_
	Rfs ^b^	^c^	FMF; BCUTp-1l; BCUTw-1h; khs.sF; Weta1.unity; VCH-7; HybRatio; FPSA-1; BCUTc-1h; LOBMAX	η; Parr & Pople absolute hardness; ω; χ; ε_LUMO_; Schuurmann MO shift alpha; ε_HOMO_; μ	khs.sssCH; MDEO-12; XlogP; TopoPSA; MDEO-11; VC-6; FMF; ATSc5; VCH-7; VC-5
		^d^	Weta1.unity; MDEC-33; TPSA; ATSc3; khs.sssCH; Weta2.unity; TopoPSA; PPSA-3; geomShape; ALogp2	ε_HOMO_; ε_LUMO_; ω ; η; Parr & Pople absolute hardness; χ; Schuurmann MO shift alpha; μ	TopoPSA; MDEO-12; ATSc1; FPSA-2; khs.sssCH; XlogP; RNCG; nHBAcc; AMR; DPSA-2
	CT		**8D**: SP-6; BCUTc-1h; Weta1.unity; Wnu1.unity; MDEC-11; SC-5; VP-7; MDEC-22	**3D**: ε_HOMO_; Parr & Pople absolute hardness; ε_LUMO_	**11D**: SP-6; ε_HOMO_; BCUTc-1h; Wnu1.unity; FNSA-3; THSA; Wlambda2.unity; MDEC-33; FPSA-3; ATSp5; ω
**Antitumor activity**	SVM		**42D**: ALogp2; AMR; BCUTw-1h; BCUTp-1l; PNSA-3; FPSA-3; FNSA-2; WNSA-3; THSA; TPSA; naAromAtom; nAromBond; ATSc2; ATSc3; ATSc4; ATSc5; bpol; C1SP2; C2SP2; SCH-4; SCH-5; VCH-4; VCH-7; VC-6; SPC-5; FMF; HybRatio; khs.dsCH; khs.aaCH; khs.sssCH; khs.tsC; khs.sNH2; khs.dO; khs.ssO; khs.sF; MDEC-12; MDEC-13; MDEC-22; MDEO-11; MDEO-12; MDEO-22; TopoPSA	**8D**: ε_HOMO_; ε_LUMO_; χ; Parr & Pople absolute hardness; Schuurmann MO shift alpha; η; μ; ω	**44D**: ALogp2; AMR; BCUTw-1h; BCUTp-1l; PNSA-3; FPSA-3; FNSA-2; WNSA-3; THSA; TPSA; Wnu1.unity; naAromAtom; nAromBond; ATSc2; ATSc3; ATSc4; ATSc5; bpol; C1SP2; C2SP2; SCH-4; SCH-5; VCH-4; VCH-7; VC-6; SPC-5; FMF; HybRatio; khs.dsCH; khs.aaCH; khs.sssCH; khs.tsC; khs.sNH2; khs.dO; khs.ssO; khs.sF; MDEC-12; MDEC-13; MDEC-22; MDEO-11; MDEO-12; MDEO-22; TopoPSA; ε_HOMO_
	Rfs	^c^	khs.sssCH; BCUTp-1l; TopoPSA; VC-5; FMF; MDEO-12; VC-6; RNCS; VCH-7; BCUTw-1l	Parr & Pople absolute hardness; η; ε_LUMO_; ε_HOMO_; χ; Schuurmann MO shift alpha; μ; ω	khs.sssCH; MDEO-12; XlogP; TopoPSA; MDEO-11; VC-6; FMF; ATSc5; VCH-7; VC-5
		^d^	TopoPSA; MDEO-12; ATSc1; khs.sssCH; XlogP; AMR; FPSA-2; MDEO-11; nHBAcc; Weta3.unity	ε_HOMO_; ε_LUMO_; η; Parr & Pople absolute hardness; ω; χ; μ; Schuurmann MO shift alpha	TopoPSA; MDEO-12; ATSc1; FPSA-2; khs.sssCH; XlogP; RNCG; nHBAcc; AMR; DPSA-2
	CT		**9D**: MDEO-12; Khs.sssCH; MDEO-11; SCH-7; nAromBond; VC-6; BCUTc-1h; C2SP2; BCUTp-1l	**4D**: ε_HOMO_; Parr & Pople absolute hardness; ε_LUMO_; χ;	**9D**: MDEO-12; Khs.sssCH; MDEO-11; SCH-7; nAromBond; VC-6; BCUTc-1h; C2SP2; BCUTp-1l
**Antibiotic activity**	SVM		**41D**: ALogP; BCUTw-1h; BCUTp-1l; DPSA-3; FPSA-3; RPCG; RNCS; TPSA; Wnu1.unity; nAromBond; ATSc1; ATSc3; ATSc5; ATSm1; ATSm5; nBase; C2SP2; C3SP3; SCH-4; SCH-5; VCH-4; VCH-7; SC-4; SC-5; VPC-5; nHBAcc; khs.sssCH; khs.tsC; khs.dssC; khs.sOH; khs.ssO; khs.sF; nAtomLC; MDEC-13; MDEC-22; MDEO-11; MDEO-12; MDEO-22; MOMI-XZ; TopoPSA; XlogP	**8D**: ε_HOMO_; ε_LUMO_; χ; Parr & Pople absolute hardness; Schuurmann MO shift alpha; η; μ; ω	**42D**: ALogP; BCUTw-1h; BCUTp-1l; DPSA-3; FPSA-3; RPCG; RNCS; TPSA; Wnu1.unity; nAromBond; ATSc1; ATSc3; ATSc5; ATSm1; ATSm5; nBase; C2SP2; C3SP3; SCH-4; SCH-5; VCH-4; VCH-7; SC-5; VPC-5; nHBAcc; khs.sssCH; khs.tsC; khs.dssC; khs.sOH; khs.ssO; khs.sF; nAtomLC; MDEC-13; MDEC-22; MDEC-24; MDEO-11; MDEO-12; MDEO-22; MOMI-XZ; TopoPSA; XlogP; ε_HOMO_
	Rfs	^c^	MDEC-22; MDEO-12; C2SP2; TopoPSA; khs.dsCH; khs.sssCH; FMF; VC-5; C4SP3; MDEC-33	Parr_&_Pople_absolute_hardness; η; ε_LUMO_; ε_HOMO_; χ; Schuurmann MO shift alpha; μ; ω	MDEC-22; MDEO-12; C2SP2; khs.sssCH; khs.dsCH; TopoPSA; khs.dssC; MDEC-33; FMF; C4SP3
		^d^	TopoPSA; ATSc1; FPSA-2; MDEC-22; MDEO-12; RNCG; C2SP2; VC-5; khs.sssCH; khs.dssC	ε_HOMO_; ε_LUMO_; η; Parr & Pople absolute hardness; ω; χ; μ; Schuurmann MO shift alpha	TopoPSA; ATSc1; FPSA-2; RNCG; MDEO-12; MDEC-22; nHBAcc; khs.sssCH; C2SP2; khs.dssC
	CT		**16D**: TopoPSA; C2SP2; VC-5; MDEC-22; XlogP; BCUTp-1h; VP-0; SCH-7; DPSA-1; Khs.dssC; MDEC-12; Khs.sssCH; THSA; MDEO-12; C2SP3; HybRatio	**3D**: ε_HOMO_; Parr & Pople absolute hardness; ε_LUMO_	**16D**: TopoPSA; C2SP2; VC-5; MDEC-22; XlogP; BCUTp-1h; VP-0; SCH-7; DPSA-1; Khs.dssC; MDEC-12; Khs.sssCH; THSA; MDEO-12; C2SP3; HybRatio

Notes: ^a^ The selection of the descriptors was with the CFS (correlation-based feature subset selection) filter from Weka; ^b^ The mean decrease in accuracy and the mean decrease in Gini are two measures of importance for the descriptors using the Rf; ^c^ MeanDecreaseAccuracy; ^d^ MeanDecreaseGini.

Furthermore, the ε_HOMO_ is the most important descriptor for MeanDecreaseGini measure in the PM6 RF model and it is as well selected to build the CDK+PM6 SVM model (see Table 5). Using the first rule of the overall biological activity classification tree PM6 (ε_HOMO_ ≥ −10.42 eV for active compounds) it was possible to correctly discriminate as active 1577 compounds, corresponding to ~98% of the training set, 774 compounds, corresponding to ~97% of the test set I, and 181 compounds, corresponding to ~99% of the test set II. It is known that the ε_HOMO_ and the energy of the lowest unoccupied molecular orbital (ε_LUMO_) of a molecule play important roles in intermolecular interactions [34], through the interaction between the ε_HOMO_ of the drug with the ε_LUMO_ of the receptor and *vice versa*. Increasing ε_HOMO_ and decreasing ε_LUMO_ in the drug molecule lead to enhancement of stabilizing interactions, and consequently, binding with the receptor [34]. The remarkable performance of this descriptor in discriminating lead-like or drug-like compounds has never been reported, however it was reported that the ε_HOMO_ could be used to predict the potency of H2 blockers such as famotidine, ranitidine, and nizatidine and the authors found a positive correlation between the ε_HOMO_ and the potency of the drugs [35]. As can be seen in Table 4, the performance of the PM6 Rf model is even better than the CDK+PM6 Rf model for the test set II with a G-mean of 0.90 and 0.81, respectively.

**Scheme 1:**
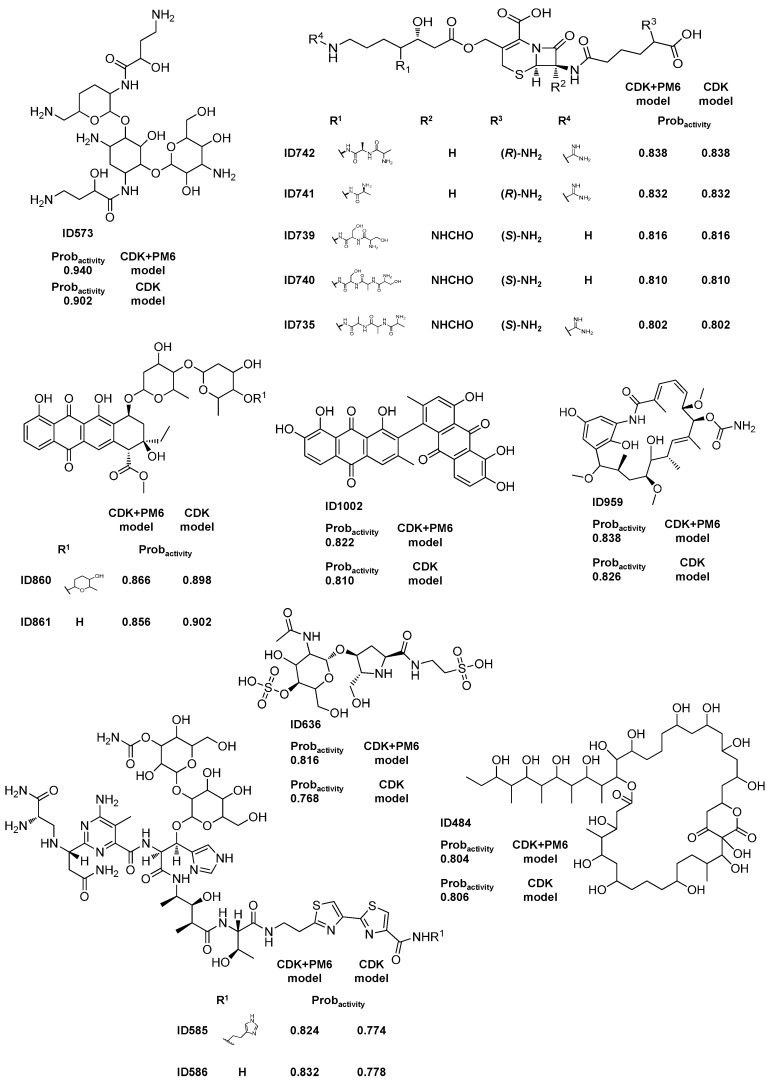
Fourteen selected lead bioactive MNPs and M_b_NPs from the AntiMarin database using the best Rf activity model.

Fourteen MNPs and M_b_NPs (Scheme 1) were listed as having a probability of being active (Prob_activity_) greater than or equal to 0.8 using the best Rf activity model. For the test set I, only active compounds were predicted with a Prob_activity_ greater than or equal to 0.8 using the best Rf activity model. We propose these MNPs and M_b_NPs as lead bioactive compounds and consider that they have been misclassified as non-active compounds in the AntiMarin database. 

Additionally, eleven out of the fourteen proposed lead bioactive compounds were also proposed by us using a CDK Rf model [15]. The other three FPs were proposed as lead antibiotic compounds in the Rf antibiotic model using the CDK descriptors and they were predicted as active with a Prob_activity_ greater than or equal to 0.77 in our previously CDK Rf overall biological activity model [15].

Interestingly, only one compound, the aklavinone glycoside (ID 860) of those fourteen proposed MNPs and M_b_NPs lead bioactive compounds has a ε_HOMO_ value lower than −10.42 eV. Some of these lead bioactive MNPs and M_b_NPs from AntiMarin database were more recently reported as being active in the literature. For instance, the 1-*N*-acyl derivative of the arbekacin (ID 573 see Scheme 1), was identified as being resistant to many inactivating enzymes while retaining most of the intrinsic antibiotic activity of the unsubstituted molecules against susceptible strains [36]. Bleomycins are currently known as a complex of related glycopeptide antibiotics produced by the bacterium *Streptomyces verticillus* [37] and are clinically used for treatment of certain cancer types [38]. As far as we know the specific bioactivities of the bleomycin derivatives (IDs 585 and 586) from AntiMarin database have never been recorded, although the *N*-(3-methylsulfinyl)propyl bleomycin is employed clinically in combination with a number of other agents for the treatment of several types of tumors, e.g., notably squamous cell carcinomas and malignant lymphomas [38].

#### 2.1.2. Antitumor Activity Model

**Scheme 2:**
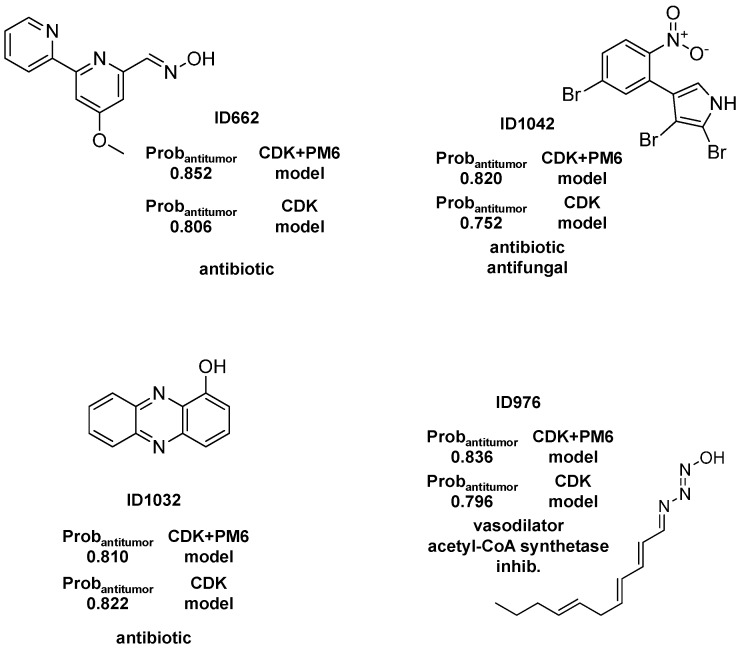
The selected 4 lead antitumor MNPs and M_b_NPs from the AntiMarin database using the best Rf antitumor model.

**Scheme 3:**
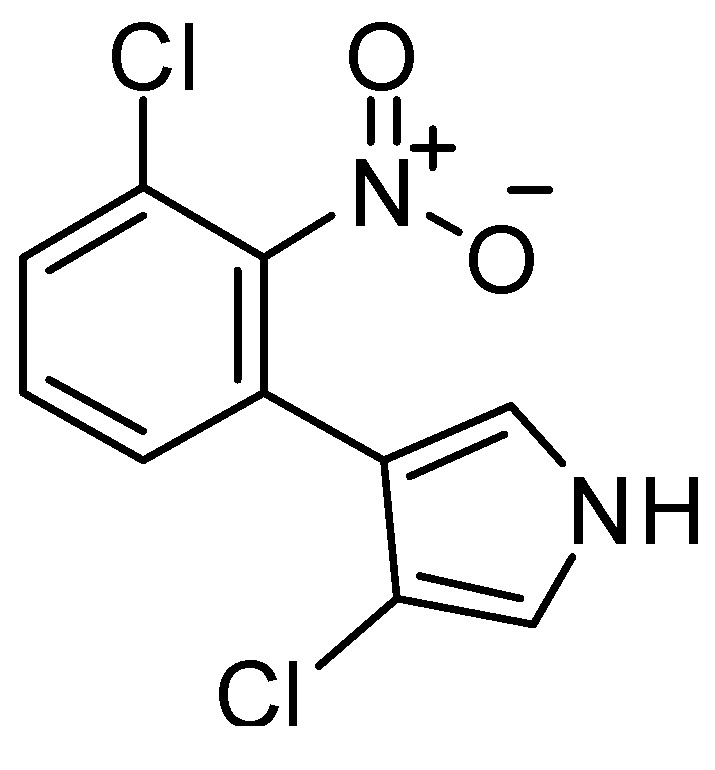
The chlorinated pyrrolnitrin from the test set (CID 13916).

For the best antitumor model (CDK+PM6 Rf model) we obtained a large number of FPs as well as FNs for the AntiMarin set, as revealed by the low sensitivity and specificity values of 0.31 and 0.71, respectively. These values could be compared with the sensitivity and specificity values of 0.85 and 0.81 obtained for the test set I, respectively Table 2. The analysis of the predictions obtained for the AntiMarin set has shown that there are 18 TPs with an average probability of being antitumor (Avg.Prob_antitumor_) of 0.60, as compared with an Avg.Prob_antitumor_ of 0.62 obtained for the 104 FPs predicted. In our opinion, these FPs are not all misclassifications by the antitumor model. Instead those with high probability of being antitumor (Prob_antitumor_) are lead-like antitumor MNPs and M_b_NPs, which have been misclassified as non-antitumor compounds in the AntiMarin database. Although, there was obtained a high sensitivity value of 0.82 for the external test set II, was also achieved a low specificity value of 0.23. For the test set II were used the biological activity reported in the pharmacological classification of each API. However for this data set in the PubChem were reported an average value of 445 tested bioassays and for those an average value of 43 active bioassays. Therefore, we obtained 28 TPs and 46 FPs with a Prob_antitumor_ greater than or equal to 0.8. The analysis of the eleven FPs with higher Prob_antitumor_ (*i.e.*, Avg.Prob_antitumo_r of 0.93) has shown that they all have at least one positive bioassay against cancer (available as Supplementary Material—Table S5). These conclusions were also supported by the low percentage of misclassifications as FPs (1.04%) obtained for the test set I compounds with a Prob_antitumor_ greater than or equal to 0.8 using the best Rf antitumor model. In the Scheme 2 were listed the 4 FPs with a Prob_antitumor_ greater than or equal to 0.8 using the best Rf antitumor model. Two out of the four proposed lead antitumor compounds were also proposed by us using a CDK Rf model in our recently published work [15]. The other two FPs were similarly predicted as antitumor with a Prob_antitumor_ greater than or equal to 0.75 in the CDK Rf antitumor activity model [15]. Moreover, there are eight (IDs 78, 163, 449, 670, 918, 974, 1070, and 1102) out of sixteen proposed as lead antitumor MNPs and M_b_NPs from the AntiMarin database in our previous work that are not present in the currently study. The other five lead antitumor compounds (IDs 567, 580, 1066, 1071, and 1100) that had been recently highlighted in our studies are also predicted as antitumor compounds by the best Rf model with a Prob_antitumor_ of 0.78, 0.71, 0.55, 0.63, and 0.68, respectively. However, antitumor activity was reported more recently, for almost all of the 4 FPs that we obtained, namely (see Scheme 2): the phenazine derivatives ID 1032 as a light-activated tumor cytotoxic compound [39], the oxime derivative ID 662 against several NCI human tumor cells [40], and the nitrosohydrazone derivative ID 976 enhances the etoposide-induced cell death of human glioma cells through a synergistic effect with antitumor drugs by acting as inhibitor of acetyl-coenzyme A synthetase [41]. The antifungal brominated phenylpyrrole derivative, ID 1042 (Scheme 2), is related to the chlorinated pyrrolnitrin, an antibiotic and antifungal MNP from test set I (CID 13916, see Scheme 3) that was recently reported as active in screenings for potential anti-tumor activity [42].

Moreover both phenylpyrrole derivatives (*i.e.*, ID 1042 from AntiMarin set and CID 13916 from test set I) were predicted as being antitumor by the Rf model with a Prob_antitumor_ of 0.82 and 0.90, respectively. With respect to FNs, the analysis of the predictions obtained for the AntiMarin set has shown that there are 256 TNs with an Avg.Prob_antitumor_ of 0.25, compared with an Avg.Prob_antitumor_ of 0.28 obtained for the 40 FNs predicted. From these 40 FNs only six M_b_NPs were predicted with a Prob_antitumor_ lower than 0.15. Interestingly four of these compounds (IDs 447, 448, 542, and 862) belong to the class of anthracycline antibiotics, one of the most widely used classes of antitumor antibiotics.

#### 2.1.3. Antibiotic Activity Model

**Scheme 4:**
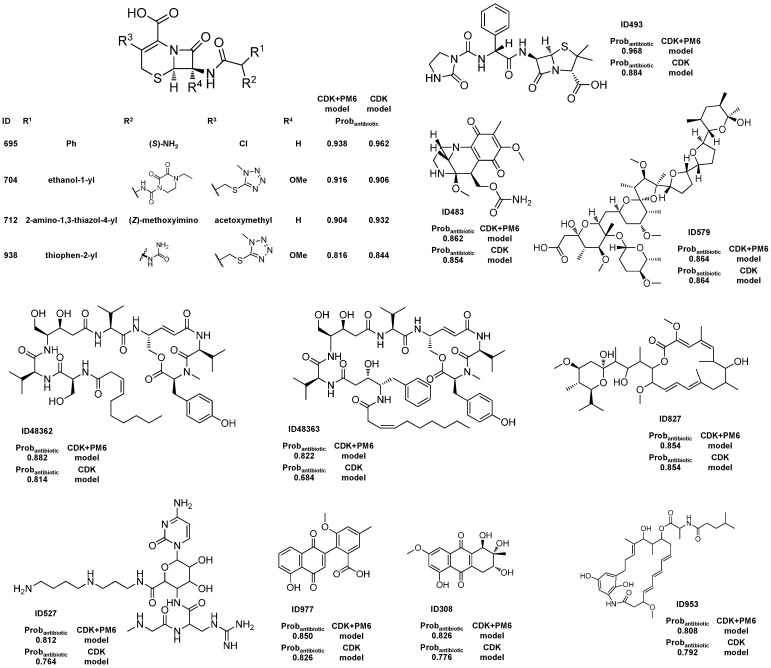
The unreported 15 lead antibiotic MNPs and M_b_NPs from AntiMarin database using the best Rf antibiotic model with a Prob_antibiotic_ greater than or equal to 0.8.

The best antibiotic model (CDK+PM6 Rf model) yielded a large number of FPs as well as FNs for the AntiMarin set, as shown by the low sensitivity and specificity values of 0.71 and 0.63 obtained, respectively. These values could be compared with the sensitivity and specificity values of 0.82 and 0.94 obtained, respectively, for the test set I, Table 3. The analysis of the predictions obtained for the AntiMarin set has shown that there are 161 TPs with an average probability of being antibiotic (Avg.Prob_antibiotic_) of 0.83, as compared with an Avg.Prob_antibiotic_ of 0.72 obtained for the 70 FPs predicted. In our opinion, these FPs are not all misclassifications by the antibiotic model but instead those with high probability of being antibiotic (Prob_antibiotic_) may be lead-like antibiotic MNP and M_b_NP, which have been misclassified as non-antibiotic compounds in the AntiMarin database. As we had done for the antitumor and biological overall models, we proposed 25 FPs with a Prob_antibiotic_ greater than or equal to 0.8 for the AntiMarin set as lead antibiotic compounds Table S1, given in Supplementary Material. The analysis of the predictions obtained for AntiMarin set has shown that there are seven FPs with a Prob_antibiotic_ greater than or equal to 0.9. From those seven FPs, four FPs (IDs 585, 586, 573, and 860) have been already analyzed in the overall biological model (Scheme 1). The other three FPs (IDs 695, 704, and 712) were reported before by us [15] as lead-like antibiotic compounds with a Prob_antibiotic_ greater than or equal to 0.9. Additionally, twenty one out of the twenty five proposed lead antibiotic compounds were as well proposed by us using the CDK Rf model [15]. The other four FPs (IDs 308, 527, 953, and 48363) were classified as antibiotic compounds in the Rf antibiotic model using the CDK descriptors and they were predicted as antibiotic with a Prob_activity_ greater than or equal to 0.68 in the CDK Rf overall biological activity model [15]. From those 25 lead-like antibiotic MNP and M_b_NP, seven (IDs 484, 735, 742, 861, 959, 739, and 741) have been already analyzed in the overall biological model (Scheme 1). Scheme 4 illustrates the 15 FPs with a Prob_antibiotic_ greater than or equal to 0.8, which have not yet been reported in this work. As recently described from our team [15], several cephalosporin analogs that appear in the AntiMarin database without activity records, were classified as actives by the overall activity and antibiotic models indicating that the cephalosporin core structure appears to be relevant to the antibiotic activity. The structures could be seen in Scheme 1 (IDs 735, 739, 741, and 742) and Scheme 4 (IDs 695, 704, 712 and 938). However, it is not a surprising outcome, since penicillin and cephalosporin are well known antibiotic drugs and the earliest antibiotics discovered. A penicillin analog was also classified as FP, ID 493 in Scheme 4. Among other relevant types of antibiotics that were classified as FPs in both models are the aminoglycoside (ID 573) and the anthracycline antibiotics (IDs 860 and 861). Two unique cyclic peptides that incorporate unusual λ-amino acids such as 4-amino-5-hydroxypenta-2-enoic acid, 4-amino-3,5-dihydroxypentanoic acid, and 4-amino-3-hydroxy-5-phenylpentanoic acid (IDs 48362 and 48363) were also classified as being antibiotic. As far we know the antibiotic activity of these compounds has never been recorded, but they were reported as shown immunosuppressive activity in an interleukin-5 production inhibition assay [43].

Three large macrocyclic lactone and lactam ring derivatives (IDs 484, 827, 953) were also classified as being antibiotic. Although the lack of reported antibiotic activity for these derivatives, the antibiotic activity of the macrolides (a class of compounds with a large macrocyclic lactone ring) is well known.

### 2.2. Analysis of Empirical and Quantum Descriptors Identified as Relevant for Modeling Overall Biological Activity, Antitumor and Antibiotic Activities

The sets of descriptors selected by the CFS filter to develop the models were presented in Table 5. All CDK+PM6 models were built using CPSA (charge partial surface area) [44], topological, constitutional, molecular descriptors, and semi-empirical quantum-chemical descriptors. The TopoPSA—topological polar surface area—descriptor [45] was selected to be used in all models. The models were built using three approaches, one with 232 CDK descriptors, the other with the 8 semi-empirical quantum-chemical descriptors calculated by the PM6 method and with the 232 CDK and the 8 PM6 descriptors. The selected and the most important descriptors obtained with these three approaches were compared with our previous studies [15] using only the CDK descriptors. The ten most relevant descriptors, found by the Rf algorithm, used to build the Rf models also include in almost cases the different types of descriptors mentioned above. The MeanDecreaseAccuracy parameter (Mean Decrease in Accuracy) of importance is considered more reliable than the MeanDecreaseGini parameter (Mean Decrease in Gini Coefficient) [46]. Taking into account the MeanDecreaseAccuracy measure in the best Rf model for predicting the overall biological activity, antitumor and antibiotic activities it is possible to correlate those activities with the type of descriptors selected. For instance, the overall biological activity appears to be related with the electronic, molecular and quantum descriptors. The CPSA, BCUT and WHIM descriptors as well as the ε_HOMO_ seem to have a main role in modeling the overall activity. The BCUT descriptors encode connectivity information and atomic properties of the molecule [47]. The WHIM descriptors are weighted holistic invariant molecular descriptors that are built in such a way to capture relevant molecular 3D information with respect to molecular size, shape, symmetry and atom distribution [48]. Nevertheless, the antitumor activity appears to be largely related with the topological descriptors. In addition, the molecular distance edge descriptors (MDE), which evaluate molecular distance edge descriptors for carbon, nitrogen and oxygen atoms, seems to be the most important type of the descriptors for modeling the antitumor activity. Only the MDEO-12 (molecular distance edge between primary and secondary oxygen atoms) and the khs.sssCH (a fragment count descriptor that encode the presence of a tertiary carbon group in which it has three single bonds) descriptors were selected for all machine learning techniques (*i.e.*, CFS filter for SVM, the two measures of the Rf, and CT using the CDK+PM6 approach). 

The MDEO-12 is the second most important descriptors for the MeanDecreaseAccuracy and MeanDecreaseGini parameters in the best Rf model and it is as well the first descriptor selected to build the antitumor classification tree. Using the first rule of the antitumor tree (MDEO-12 < 0.999 for antitumor compounds and MDEO-12 ≥ 0.999 for non-antitumor compounds) it was possible to correctly discriminate antitumor/non-antitumor 722/500 compounds, corresponding to ~70% of the training set and 355/233 compounds, corresponding to ~68% of the test set I. The results were even impressive when only the “MDEO-12 < 0.999” rule was applied—from the 880 antitumor compounds 722 were correctly classified, ~82%, for the training set and 355 out of 438, ~81%, for the test set I. All the lead-like antitumor MNPs and M_b_NPs that we had already proposed are classified as antitumor by this rule. The remarkable performance of this descriptor in discriminating between antitumor and non-antitumor compounds was reported before by us [15]. The MDEO-12 descriptor is known to codify the molecular size by taking into account oxygen atoms also characterizes polarity [49]. Inspection of the compounds belonging to the training set reveals that this descriptor provides an indication of the presence of oxygen-containing groups such as glycosyl, amide, lactam, ester or lactone together with hydroxyl, carboxylic acid or ether functional groups. 

The antibiotic activity seems to be related mainly with the topological and constitutional descriptors. Four descriptors were selected for all machine learning techniques—one electronic (TopoPSA), two topological (MDEO-12 and MDEC-22—molecular distance edge between secondary carbon atoms), and one constitutional (khs.sssCH). The TopoPSA is the most important descriptor for the MeanDecreaseGini parameter in the Rf model and it is as well the first descriptor selected to build the antibiotic tree. Using the first rule of the antibiotic tree (TopoPSA ≥ 120.7 for antibiotic compounds and TopoPSA < 120.7 for non-antibiotic compounds) it was possible to correctly discriminate 499/854 and 256/394 antibiotic/non-antibiotic compounds for the training set (642/1104) and test set I (326/537), respectively. Subsequently the TopoPSA descriptor presents similar results for both antibiotic and non-antibiotic classes differently to the antitumor CT model where the MDEO-12 descriptor shows impressive discrimination ability only relatively to the antitumor compounds. Globally 1353 out of 1746 compounds, ~77%, were correctly classified by this rule with 499 out of 642 antibiotic compounds, ~78%, and 854 out of 1104 non-antibiotic compounds, ~77%, correctly classified for the training set. Similar results were obtained for the test set I. Only two M_b_NP of 25 lead-like antibiotic MNPs and M_b_NPs that we had already proposed are classified as non-antibiotic (IDs 483 and 977) by this rule. The remarkable of this descriptor in discriminating between antibiotic and non-antibiotic compounds is in accordance with our previous study [15].

## 3. Experimental Section

### 3.1. Data Sets and Descriptors

The training and test sets I were extracted from the PubChem database [50,51], searching by the different types of biological activity available in the database (e.g., antitumor, antibiotic, antimicrobial, antifungal, antimalarial, anti-HIV). The retrieved chemical structures were saved as SMILES strings. The training and the test sets are the same which had been used to build the QSAR models, for classification, of our previous work [15] (the data set was randomly partitioned in training and test set), with exception of a few compounds that produce error in the process for calculation of the semi-empirical quantum-chemical descriptors. The training set and test set I consists of 1318 antitumor, 968 antibiotic, 55 antifungal, 11 antimalarial, one anti-HIV, 74 anti-microbial, 145 cytotoxic and 199 non-active compounds. Of note some compounds have more than one bioactivity record. The non-active compounds were selected on the basis that they were screened for at least one biological activity. The training set consists in 1746 compounds and the test set I in 863 compounds. These datasets were used for the development and external validation of the QSAR classification models, respectively. A further set, AntiMarin set, with 418 MNPs and M_b_NPs was extracted from the AntiMarin database [6,17,18]. This set will not be used to test the model but to exploit lead-like molecules from MNPs and M_b_NPs. An additional test set, test set II was also extracted from PubChem, which contains 183 APIs with the following pharmacological classifications: 68 antitumor, 29 antibiotic, eight anti-HIV, 34 anti-inflamatory, 11 antifungal, six antiviral, six insecticides, nine antimalarial, and seven anti-ulcer. The chemical structures of the MNPs and M_b_NPs were downloaded in the MDL SDF format. The different sets were assembled and duplicates removed to avoid overlapping between the two databases. Although the chirality was taken into account, racemic compounds (or cases where the stereochemistry was not indicated) were considered as one of the possible stereoisomers. The SMILES strings of the four different data sets, the natural source for the compounds of AntiMarin set, and the corresponding experimental and predicted activities are available as Supplementary Material Tables S2–S5.

### 3.2. Molecular Descriptors

JChem Standardizer tool version 5.12.3 (ChemAxon Ltd., Budapest, Hungary) were used to standardize the molecular structures by normalization of tautomeric groups and by removing of small fragments. Then CORINA version 2.4 (Molecular Networks GmbH, Erlangen, Germany) were used to generate 3D structures of the compounds. From the 3D structures 270 empirical molecular descriptors, including electronic, topological, geometrical, constitutional, and hybrid (BCUT and WHIM) descriptors, were calculated using the CDK Descriptor Calculator 1.3.2 [52,53].

The calculation of the semi-empirical quantum chemical descriptors was performed in a semi-automatic way using the following steps: (a) generation of the most stable conformer using the leconformer method, with JChem CXCALC tool (ChemAxon Ltd.), (b) optimization of the 3D geometry with MOPAC2009 [54] using the PM6 semi-empirical method [55], and (c) calculation of the harmonic vibrational frequencies to determine if the optimized geometry was minima on the potential energy surface (all real frequencies) at the same theory level. The semi-empirical quantum chemical descriptors extracted directly from the MOPAC output were the energy of the highest occupied molecular orbital, ε_HOMO_, and the energy of the lowest unoccupied molecular orbital, ε_LUMO_. From these two orbital energies the following descriptors were calculated: hardness, η = (ε_LUMO_ − ε_HOMO_); chemical potential, μ = −(ε_HOMO_ + ε_LUMO_)/2; Mulliken electronegativity, χ = −μ; Parr & Pople absolute hardness, (ε_HOMO_ − ε_LUMO_)/2; Schuurmann MO shift alpha, (ε_HOMO_ + ε_LUMO_)/2; electrophilicity index, ω = μ^2^/(2η) as defined by Parr *et al.* [56]. The quantum-chemical descriptors were measured in electronvolt (eV).

### 3.3. Selection of Descriptors and Optimization of QSAR Classification Methods

The first step for the selection of the best set of descriptors to model the different activities was the removal of constant descriptors (*i.e.*, a descriptor that presents the same value for all compounds of a data set, this usually being zero). After that, multilinear regressions (MLR) were built with Weka 3.6.5 [57,58,59] to select descriptors by the M5 method, using the training set which give origin to a set 232 descriptors. The next step in the selection of the best set of descriptors to model each activity consists in the application of the Correlation-based Feature Subset Selection algorithm [60] (the algorithm evaluates the usefulness of individual descriptors for predicting the given activity and also the level of intercorrelation among descriptors) implemented in Weka 3.6.5. The selection was performed using the AttributeSelectedClassifier routine of Weka with the CfsSubsetEval option as descriptor evaluator and the BestFirst, LinearForwardSelection or GreedyStepwise option as search methods within a ten-fold cross-validation procedure and *k* nearest neighbor (KNN) algorithm as ML technique.

### 3.4. ML Techniques

The KNN algorithm [61] predicts the activity for a compound by majority voting of the *k* most similar compounds in the training set. The KNN models were implemented with the Weka 3.6.5 software using as parameters a *k* of 10 (10 most similar neighbours of the query compound), Euclidean distances (as measure of similarity), and contributions of neighbours weighted by the inverse of distance.

CTs were grown using the RPART library, with the default parameters [62,63], in R program, version 2.13.1. A classification tree is sequentially constructed by partitioning compounds from a parent node into two child nodes. Each node is produced by a logical rule defined for a single descriptor, where objects below a certain descriptor’s value fall into one of the two child nodes, and objects above fall into the other child node. The prediction for a compound reaching a given terminal node is obtained by a majority vote of the objects (in the training set) reaching the same terminal node. The CT models were built by three approaches, one using 232 CDK descriptors, the other using eight semi-empirical quantum-chemical descriptors calculated by the PM6 method (PM6 descriptors) and finally using both the 232 CDK descriptors and the 8 PM6 descriptors.

A Rf [64] is an ensemble of unpruned classification trees created by using bootstrap samples of the training data set. The best split at each node was defined among a randomly selected subset of descriptors. Prediction is made by a majority vote of the classification trees in the forest. The performance is internally assessed with the prediction error for the objects left out in the bootstrap procedure (internal cross-validation or OOB estimation). In addition, the method quantifies the importance of a descriptor by the increase in misclassification occurring when the values of the descriptor are randomly permuted, correlated with the mean decrease in accuracy parameter, or by the decrease in a node’s impurity every time the descriptor is used for splitting, correlated with the mean decrease in the Gini coefficient parameter. Rfs also assign a probability to every prediction on the basis of the number of votes obtained by the predicted class. A measure of similarity between two objects can be calculated from the number of trees in the ensemble that classify the two objects in the same terminal node. Rfs were grown with the R program, version 2.13.1, using the Random Forest library [63]. The Rf models were built with 500 trees and by three approaches, one using 232 CDK descriptors, the other using 8 semi-empirical quantum-chemical descriptors calculated by the PM6 method (PM6 descriptors) and finally using both the 232 CDK descriptors and the 8 PM6 descriptors. As a result of the nature of two-class imbalance in the overall activity model, this problem was alleviated setting the class weights to 50:50.

SVM map the data into a hyperspace through a nonlinear mapping (a boundary or hyperplane) and then separate the two classes of compounds in this space. The boundary is positioned using examples in the training set that are known as the support vectors. With nonlinear data, kernel functions can be used to transform it into a hyperspace where the classes become linearly separable. In the present work, SVM models were established with the Weka 3.6.5 program, using the LIBSVM package [65,66,67,68]. The type of SVM was set to C-SVM-classification and the kernel function was the radial basis function. The parameter C of the C-SVM-classification was optimized in the range of 1–500 and the default γ parameter in the kernel function was used. The descriptors selected by the CFS procedure were normalized and used to develop the classification models. The active and non-active classes of the overall activity model were set to the weights of 10:90, respectively.

## 4. Conclusions

In this work, we have compared the predictive power of QSAR classification models based on quantum-chemical descriptors computed through the PM6 method and empirical descriptors such as CDK. The results suggest that the implemented computer-aided approach using quantum-chemical descriptors could be used to predict the bioactivity of new, or existing NPs without bioactivity records, and by this way identify and propose lead compounds *en route* to a specified activity with an improve in performance as compared to the QSAR model built only with CDK descriptors. The result of the application of this approach is the reduction in great extent the number of compounds used in real screens and on the other hand, allows to confirm our previous previsions. The obtained results for the presented virtual screening of possible lead compounds *en route* to antitumor and antibiotic drugs were also externally supported by the publication in the literature as active compounds of some of the compounds proposed and initially classified in the AntiMarin database as non-active compounds or without activity record. The remarkable performance of the ε_HOMO_ quantum-chemical descriptor in the discrimination in large scale data sets of lead-like or drug-like compounds has never been reported. The evaluation of the discriminating power of this quantum descriptor, calculated at a quantum level, in large sets of bioactive molecules could be an interesting approach in future works.

## Supplementary Materials

Supplementary materials can be accessed at: http://www.mdpi.com/1420-3049/20/03/4748/s1.
